# Self-reported functioning among patients with ultra-rare nemaline myopathy or a related disorder in Finland: a pilot study

**DOI:** 10.1186/s13023-023-02973-2

**Published:** 2023-11-30

**Authors:** Vilma-Lotta Lehtokari, Minna Similä, Marianne Tammepuu, Carina Wallgren-Pettersson, Sonja Strang-Karlsson, Sinikka Hiekkala

**Affiliations:** 1 Folkhälsan Research Center, Helsinki, Finland; 2 Medicum, University of Helsinki, Helsinki, Finland; 3 Clinical Nutrition Unit, Internal Medicine and Rehabilitation, Helsinki University Hospital and University of Helsinki, Helsinki, Finland; 4 Department of Paediatric Neurology, New Children’s Hospital, Helsinki University Hospital, Helsinki, Finland; 5 Department of Clinical Genetics, HUS Diagnostic Center, University of Helsinki and Helsinki University Hospital, Helsinki, Finland; 6 The Finnish Association of People with Physical Disabilities, Helsinki, Finland

**Keywords:** Congenital myopathies, Cross-sectional survey, International classification of functioning (ICF), Self-reported functioning, Nemaline myopathy, Patient-Reported Outcomes Measurement Information System (PROMIS®)

## Abstract

**Background:**

Nemaline myopathy (NM) and related disorders (NMr) form a heterogenous group of ultra-rare (1:50,000 live births or less) congenital muscle disorders. To elucidate the self-reported physical, psychological, and social functioning in the daily lives of adult persons with congenital muscle disorders, we designed a survey using items primarily from the Patient Reported Outcomes Measurement Information System, PROMIS®, and conducted a pilot study in patients with NM and NMr in Finland. The items were linked to International Classification of Functioning, Disability and Health (ICF) categories.

**Results:**

In total, 20 (62.5%) out of 32 invited persons resident in Finland participated in the study; 12 had NM and 8 NMr, 15 were women and 5 men aged 19–75 years. Sixteen (80%) were ambulatory and 4 (20%) NM patients used wheelchairs. The results from the PROMIS measuring system and ICF categories both indicated that non-ambulatory patients of this study faced more challenges in all areas of functioning than ambulatory ones, but the differences were smaller in the domains measuring psychological and social functioning than in physical functioning. In addition, the COVID-19 pandemic adversely affected the functioning of non-ambulatory patients more than that of ambulatory patients. The interindividual differences were, however, noticeable.

**Conclusions:**

To our knowledge, this pilot study is the first comprehensive survey-based study of the physical, psychological, and social functioning of adult persons with nemaline myopathy or related disorders. The results indicate vulnerability of non-ambulatory patients being at higher risk to a decrease in general functioning during global or national exceptional periods. The responses also gave directions for modifying and improving the survey for future studies.

**Supplementary Information:**

The online version contains supplementary material available at 10.1186/s13023-023-02973-2.

## Background

Muscle disorders are defined by muscle weakness and impairment of exercise endurance, causing symptoms such as fatigue and sometimes pain, which in turn often have a major impact on a patient’s daily activities and participation in social and civic life [1–4]. Congenital myopathies are a group of clinically variable ultra-rare [5] muscle disorders defined based on muscle weakness and structural abnormalities seen in muscle fibres at light microscopy. In nemaline myopathy (NM), the structural abnormalities are protein aggregates called nemaline (rod) bodies seen in Gömöri trichrome-stained muscle biopsy sections. NM usually affects proximal muscles mainly, but a few families with distal forms of NM have also been described. NM and related myopathies (NMr) vary clinically from very severe, neonatally lethal forms to those causing mild, generalised muscle weakness [6, 7]. Weakness is often selective. In the typical form of NM, weakness of the proximal muscles usually dominates the initial clinical picture, later accompanied by a distal component, while severity varies from very mild to very severe. Distal forms of nebulin-caused myopathies have been reported, with or without nemaline bodies [8–15]. A new clinical classification of NM was recently published [7]. In this classification, as well as in the previous one, patients with very unusual symptoms or signs are assigned to the category of “other forms” of NM.

Of the congenital myopathies, NM is the most common [7]. To date, variants in 12 genes are known to cause NM. Most often, causative variants are found in the genes encoding nebulin (*NEB*) and α-actin (*ACTA1*). NM and related disorders may be inherited in an autosomal recessive or dominant way. The causative variant may also be de novo and sometimes mosaic. There is a large phenotypic variability: The same gene or even the same variant may cause different histological or clinical patterns in different patients, even within the same family [11, 16].

Weakness of ventilatory muscles is a common feature in all patient groups, and this is often out of proportion to the general muscle weakness, and breathing problems related to muscle weakness are easily mistaken for asthma [17–20]. Respiratory compromise is the greatest risk for this group of patients, and many require ventilatory support. Some may require nocturnal non-invasive support, while others need continuous invasive mechanical ventilation.

To our knowledge, the self-reported physical, psychological, and social functioning of adult persons with NM or related disorder has not previously been studied. The existing studies objectively investigate a person’s functioning using physical, psychological, or cognitive tests [21–23], and some investigate the subjective experience of the person and are targeted to paediatric patients and their parents [24, 25]. In addition, some survey studies have been published regarding the quality of life, fatigue, and functional impairments of persons with neuromuscular diseases [1, 2, 26]. Comparing congenital myopathies such as NM and NMr with progressive disorders, disorders with deviant symptoms or with neurological disorders is however, difficult, because of the different natures of these disorders.

We conducted a cross-sectional pilot study in Finland to investigate how the patients with NM and NMr in Finland, themselves, experienced their physical, psychological, and social functioning and to test the survey designed for this purpose mainly utilizing the previously validated Patient-Reported Outcomes Measurement Information System (PROMIS®) item bank [27–29] as a source of items utilizing International Classification of Functioning (ICF) [30, 31]. The survey also addressed the influence of the COVID-19 pandemic.

It is necessary to study the patients’ own experiences of their functioning for better and more efficiently focused medical care and rehabilitation services.

## Methods

### Recruitment of the patients

**Fig. 1:**
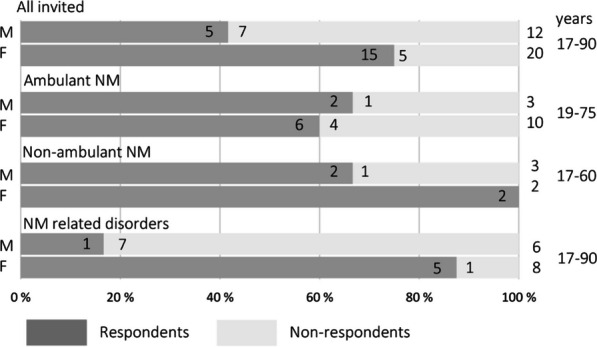
Invited patients: ambulatory nemaline myopathy (NM), non-ambulatory NM patients, and patients with NM related disorders; participants and non-responders, and the age range of the invited persons. *M* males, *F* females

We invited all 32 NM and NMr patients over 16 years of age (16–90) resident in Finland and included in the Folkhälsan Research Center registry (Fig. 1), which, to our knowledge, includes the vast majority of NM and NMr patients from all University Hospital districts in Finland. The invited patients were from 26 families. The information letters and consent forms to be signed were sent as letters to the home addresses of the patients.

### Questionnaire design

Where possible, the items for the survey were selected from previously published and/or validated questionnaires. The main item source was the Patient-Reported Outcomes Measurement Information System (PROMIS®) [27–29]. In addition, the National FinSote2019 Survey [32], and a survey designed for people with skeletal dysplasia in Finland (LYHTY) [33, 34] were used. The research group (RG) designed questions when suitable questions could not be found in existing surveys. These were questions about medical conditions diagnosed by a medical doctor, and questions related to mobility: walking ability on different surfaces, usage of mobility aids or wheelchair. Questions concerning the impact of the COVID-19 pandemic on functioning were, also, addressed by the RG. In addition, the survey included open text fields for optional comments after each subject field. The panelists selecting the PROMIS® items were five persons with NM and RG members, following the validation procedure described in [35]. Questions from other surveys were selected by the RG.

The survey contained eight modules, each containing questions, which in turn consist of separate items (in parentheses the number of items; the source of the item): (1) Background questions (7; 4 generic (subjects characteristics), 2 FinSote2019, 1 RG), (2) Rehabilitation services, other services, aids (22; 14 FinSote2019, 4 LYHTY, 4 RG), (3) Physical functioning (22; 20 PROMIS®, 2 LYHTY), (4) Social functioning (10; PROMIS®), (5) General Health (10; PROMIS®), (6) Pain, Fatigue and Sleep (12; PROMIS®), (7) Mental well-being (5; PROMIS®) (8) Questions related to the muscle disorder and other medical conditions and treatments (12; 1 generic, 1 FinSote2019, 5 LYHTY, 5 RG).

Most items addressing the self-reported functioning were selected from validated PROMIS® questionnaires. The following PROMIS® instruments were used (in parentheses the number of items selected per instrument): Global health (Physical (5), Mental (5)), Physical function (24), Fatigue (4), Sleep Disturbance (4), Satisfaction with Social Roles and Activities (8), Pain Interference (3), Emotional Distress—Depression (3), Ability to Participate in Social Roles and Activities (2), Emotional Distress—Anger (1) and Anxiety (1).

The final questionnaire was created using Webropol, and the subjects could fill it out either electronically or on paper. Any uncertainties or discrepancies in the responses were clarified with the participant by telephone by the first author.

### Linking the items to International ICF categories and forming the sum variables

Each five-point Likert scale variable (item) was linked to an International Classification of Functioning in Disability and Health (ICF)-category [30] using the ICF linking rules [36]. For calculating the sum variables, the responses were converted as follows: point 1 indicated “no problems” and point 5 indicated “a lot of problems”/ “unable to”; in other words, a higher number indicated more poor functioning.

The ICF blocks and chapters were fitted to form sum variables to minimize the number of items. The following ICF-based categories were formed (the number of items included per category in parentheses); b1 Mental functions: fatigue (5), sleep (4), emotional distress (6), b2 Sensory functions and pain: muscle pain (3) and joint pain (1) and sensation of pain (4), b7 Neuromusculoskeletal and movement-related functions: muscle functions (2), d2 General tasks and demands: carrying out daily routine (1), d4 Mobility: changing and maintaining body position (6), hand and arm use (8), walking and moving around using equipment (3), moving around within a home (1), d4 Selfcare: dressing, toileting, washing oneself (7), d6 Domestic life: acquisition of goods and services (2), doing house work (1), leisure time at home (1), d7 Interpersonal interactions and relationships (2), d8 Major life areas: work (1), d9 Community, social and civic life, e1 Products and technologies: assistive products (1), assistive products for mobility (3), e2 Natural environment: climate (2), and the impact of Covid-19 pandemic to fatigue, sleep, emotions, pain, general health, physical functioning, daily living, social life, applying health and social services, access to health and social services. The items addressing the ability to walk and move around using equipment included the use of mobility aids (a self-propelled or electric wheelchair, or a walker). The formed variables were sorted into groups: physical, psychological, and social functioning, and environmental factors affecting physical functioning. The impact of COVID-19 pandemic to the self-reported functioning was processed as its own entirety.

The internal consistencies of the sum variables formed were tested using Cronbach’s alpha [37]. If Cronbach’s alpha was below 0.7, the item(s) not computationally belonging to the sum variable was removed from the group and analyzed separately. The Cronbach’s alphas of the final sum variables formed varied from 0.73 to 0.96.

ICF categories for the areas of functioning assessed, the source of the item used (and number of items), and Cronbach’s α-value for the sum variables are presented in Additional file 1.

### Calculating T-scores for the PROMIS instruments

PROMIS item banks contain a collection of items, each measuring the same domain. This enables a selection of single items to be used to study the domain [38]. The PROMIS instrument is based on Item Response Theory (IRT) and a software application utilizing it. Health Measures Scoring Service (HM-SS), is developed to convert the raw scores (on a Likert scale of 1 to 5) of the responses to standardized T-scores for each instrument [39]. In the PROMIS T-score metric, 50 is the mean of a relevant reference population, and the standard deviation (SD) is 10 in a reference population (usually the U.S. general population). Standardized scores for the Finnish population are lacking, and therefore the T-scores were calculated using the standard scores for the US population. A standardized T-score was calculated using a scoring service [40]. The minimum requirement of the items to calculate the T-scores for the selected instruments used in this survey was four. Subsequently, the instruments for Global health, Physical function, Fatigue, Sleep Disturbance, and Satisfaction with Social Roles and Activities were scored, while Ability to Participate in Social Roles and Activities, Emotional Distress—Anger, and Anxiety were not scored. They were processed according to their ICF category-based sum variable labelled as “emotional functioning”, as described above. PROMIS scoring system includes the levels for the functioning based on T-scores of the reference and validation populations [41]. For example, for the Physical Functioning, a T-score above 45 means “within normal limits”, 40–45 “mild decline”, 30–40 “moderate decline” and below 30 “poor” physical functioning.

### Data analysis

The responses of the study participants were analyzed using Excel and IBM SPSS Statistics 28.0.

The participants’ responses were reviewed according to groups; responses of NMr (8, 40%), ambulatory NM (8, 40%), and non-ambulatory NM (4, 20%) patients. In addition, the groups under and over 50 years of age, ambulatory (both NMr and NM patients), and NM patients (including ambulatory and non-ambulatory patients) were reviewed.

We calculated percentages of the responses of each ICF-based variable (i.e., how many percent of the responses in each group were 1 (no/never problems), 2 (little/rarely problems), 3 (some/sometimes problems), 4 (much/often problems) and 5 (very much/unable to/always problems), the mean values, and the standard deviations of the responses.

The PROMIS-based T-scores with minimum and maximum values were processed similarly.

The survey included open text boxes for background questions and for possible additional comments the participants wanted to add or raise after each module. These responses were reviewed separately, and they can be utilized for modifying the survey for possible future studies.

## Results

### Characteristics of the participants

Of the invited 32 persons, 20 (63%) from 17 families returned the survey (Fig. 1): 75% of the invited females and 42% of the males participated the study. The youngest and the eldest patients invited did not want to or could not participate in the study.

Of the 20 participants, 15 (75%) were women and five (25%) men, aged 19–75 years (Table 1). None of the participants lived in the same household, but six participants were from three families (mother and offspring). Twelve participants (60%) fulfilled the criteria of NM, muscle weakness and nemaline rods in a muscle biopsy. Eight (67%) of the NM patients were ambulatory, while four (33%) used a wheelchair. In this study, patients who had no obvious nemaline rods in their diagnostic Gömöri trichome stained muscle biopsies were designated as patients with NM-related disorders (NMr). Altogether eight (40%) had NMr; six (75%) of them had distal myopathy without nemaline bodies and two (25%) were patients with unusual symptoms and signs, such as muscle stiffness.

**Table 1:** Characteristics of the participants by their primary diagnosis and walking ability

	All	NM nonamb	NM amb	NMr
*Participants (%)*	20 (100)	4 (20)	8 (40)	8 (40)
Females (%)/males (%)	15 (75)/5 (25)	2 (50)/2 (50)	6 (75)/2 (25)	7 (88)/1 (12)
Age in years (r)	47 (19–75)	48 (22–60)	44 (19–75)	51 (26–74)
Age in years females (r)	51 (19–75)			
Age in years males (r)	37 (22–57)			
BMI (r)	25.8 (18.2–35.6)	26.9 (20.0–32.4)	25.0 (18.8–35.6)	26.3 (18.2–34.4)
*Respiratory support n (%)*				
Non-invasive nocturnal	4 (20)	3 (75)	1 (13)	0
Mechanical continuous n (%)	2 (10)	1 (25)	1 (13)	0
*Life situation n (%)*				
Student	2 (10)	0	2 (25)	0
Unemployed	1 (5)	0	0	1 (13)
Working	9 (45)	0	4 (50)	5 (63)
Disability pensioner	5 (25)	3 (75)	1 (13)	1 (13)
Old-age pensioner	3 (15)	1 (25)	1 (13)	1 (13)
*Household structure*				
Solitaire (A)	11 (4)	2 (2)	5 (2)	4 (0)
With family (A)	9 (2)	2 (2)	3 (0)	4 (0)
*Pathogenic gene variants n (%)*				
AD *TPM2*^U^	2 (10)	0	0	2 (25)
AR HOZ mis^W^, large AD^K^ or mos^S^ del in *NEB*	6 (30)	0	0	6 (75)
Other AR *NEB*^L1^	10 (50)	3 (75)	7 (88)	0
AD^L2^/de novo* ACTA1*^U^	2 (10)	1 (25)	1 (12)	0
Conditions diagnosed by a doctor** n (%)				
Asthma, other pulmonary disease or recurrent pneumonias	8 (40)	4 (100)	2 (25)	2 (25)
Joint deformities	6 (30)	2 (50)	3 (37.5)	1 (12.5)
Joint hypermobility	7 (35)	1 (25)	5 (62.5)	1 (12.5)
Joint stiffness and/or contractures	6 (30)	2 (50)	3 (37.5)	1 (12.5)
Arthritis or rheumatic disorder	6 (30)	1 (25)	4 (50)	1 (12.5)
Osteoporosis	4 (20)	3 (75)	0	1 (12.5)
Scoliosis	8 (40)	4 (100)	3 (37.5)	1 (12.5)
Depression or anxiety	3 (15)	0	1 (12.5)	2 (25)

*NM* nemaline myopathy, *NMr* nemaline myopathy related disorder, *amb* ambulatory, *nonamb* non-ambulatory, *r* range, *A* Personal assistance admitted, *AD* autosomal dominant, *AR* autosomal recessive, *HOZ* homozygous, *TPM2* beta-tropomyosin gene, *NEB* nebulin gene, *mis* missense, *mos* mosaic, *del* deletion, *ACTA1* alfa-actin 1 gene

**Self-reported. U: unpublished, W: Wallgren-Pettersson et al. 2007, K: Kiiski and Lehtokari et al. 2019, Sagath & Lehtokari et al., 2021, L1: Lehtokari et al. 2014, L2 Lehtokari et al. 2018

The majority, 16 (80%), of the participants were ambulatory, but 11 (55%) of them used a mobility aid and/or ankle orthoses/support either all the time, outdoors, or for long distances. Those who did not need mobility aids had mild forms of NM or NMr. Four (20%) used wheelchairs. Two (10%) (one ambulatory and one non-ambulatory) of the participants used continuous mechanical ventilatory and four (20%) (one ambulatory and three non-ambulatory) nocturnal non-invasive respiratory support. All non-ambulatory patients and those requiring respiratory support had NM (Table 1).

All participants’ diagnoses had been genetically identified or verified at the Folkhälsan Research Center. The pathogenic variants were in common NM genes, *NEB*, *ACTA1,* and *TPM2* (β-tropomyosin gene)*,* located in autosomal chromosomes. The NM-causing variants were either recessive compound heterozygous variants in nebulin [11], or de novo dominant [16] or dominantly inherited *ACTA1* variants. Persons with distal forms of congenital myopathy had either homozygous missense [8], dominant, or de novo mosaic [15] large deletions in *NEB*, and those with unspecified forms had dominant *TPM2* variants (Table 1). All but the variant in *TPM2* and the de novo* ACTA1* variant had been previously published [7, 10, 13–15].

All the non-ambulatory NM, three (38%) of the ambulatory NM, and one (12.5%) NMr patient had scoliosis. Osteoporosis had been diagnosed by a clinician in three (75%) of the patients using wheelchairs and in one of the older ambulatory patients. Recurrent respiratory tract infections were reported by one (25%) and asthma or other pulmonary diseases as diagnosed by a medical doctor by three (75%) non-ambulatory patients. Joint-related problems were reported by six (75%) of the ambulatory NM patients; five of them reported hypermobility and three of the five reported having additional joint problems such as diagnosed arthritis, stiffness, contractures, or joint deformities. Problems related to joints were reported by 38% of NMr and 50% of non-ambulatory NM patients. Three (15% of all) participants had a diagnosed mental disorder, such as depression or anxiety disorder. None of them were wheelchair users (Table 1). None of the participants had had a COVID-19 infection by April 2022.

### Self-reported physical functioning

**Fig. 2:**
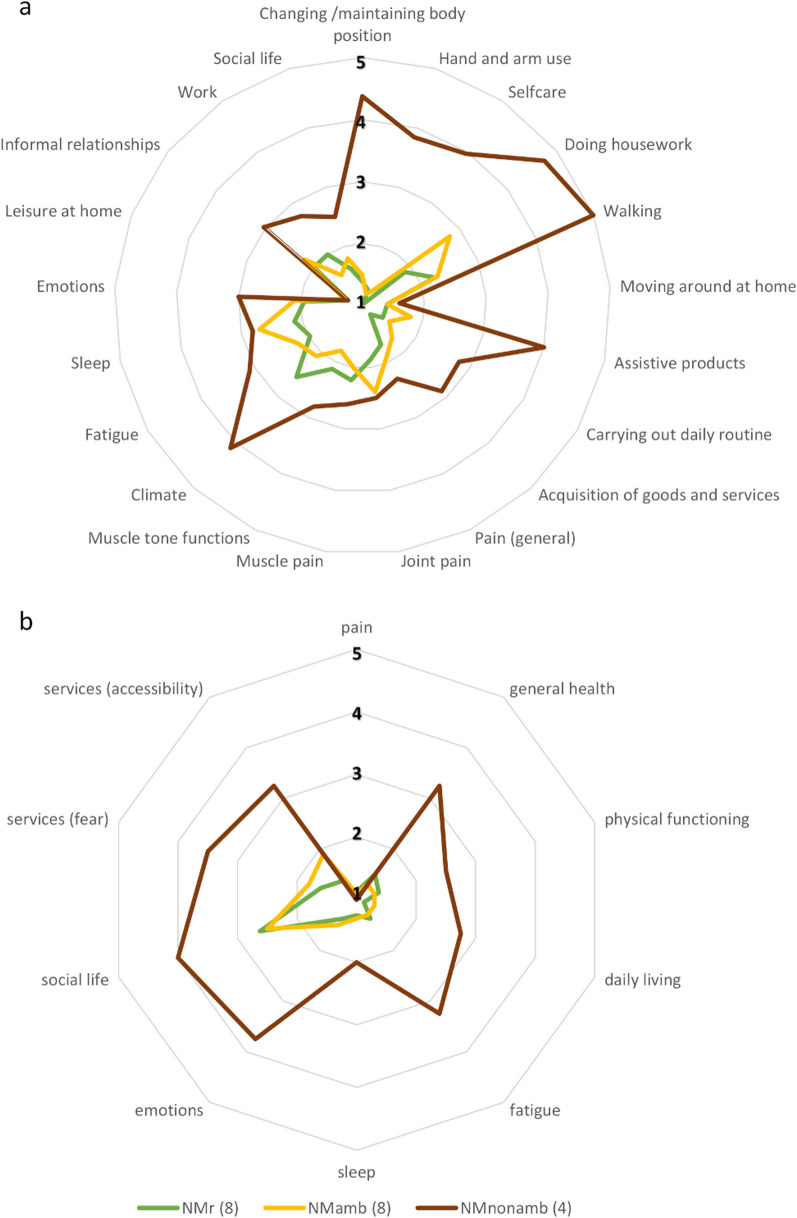
**a** The average functioning of the ambulatory and non-ambulatory nemaline myopathy patients and patients with related disorders (NMamb, NMnon-amb and NMr, respectively). Likert-scale 1 to 5 (1 = no problems/satisfied with/no need for aid or assistance, 5 = lots of problems/unable to/unsatisfied with/need for aid or assistance in everyday life). **b** The impact of COVID19 pandemics on functioning; (1 = no impact, 5 = very much negative impact). The raw, mean, and standard deviation values in Additional file 2

The largest differences between the non-ambulatory and the ambulatory participants in this study were in the categories addressing physical functioning requiring competent muscle function (e.g., self-care and doing housework, as well as hand use and walking) (Fig. 2a) The non-ambulatory participants needed more assistance in their daily tasks and many reported either great difficulties in carrying out the task in question or were unable to perform it. This was the case in changing or maintaining body position, hand and arm use, self-care (washing oneself, toileting, and dressing), and doing housework. Non-ambulatory participants faced more challenges also in the acquisition of goods and services than the others.

The self-reported physical functioning was lower in participants with NM than NMr. The biggest differences between participants with NM and NMr in mean responses were in self-care (NM 2.2 ± 1.3 and NMr 1.1 ± 0.1) and doing housework (NM 3.4 ± 1.4 and NMr 1.9 ± 0.8) (in parentheses the mean and standard deviation of responses).

Older participants found moving around within the home more difficult than younger ones, but mobility at home (with or without a mobility aid) was not considered problematic in general in this cohort (Additional file 2(a)).

Pain or muscle stiffness was, on average, not considered severe in any of the groups. The non-ambulatory NM patients experienced slightly more pain, such as muscle and joint pain, and muscle stiffness, than ambulatory NM or NMr patients. None reported having very severe muscle pain or disturbing generalized pain. One ambulatory participant with NM reported with hyperflexible joints reported having joint pain all the time, and four other participants (one non-ambulatory and three ambulatory) reported having joint pain often. All of them were over 50 years of age, obese or noted to have arthritis and/or hyperflexibility. One participant with NMr, whose primary symptom is stiffness of hand muscles, evaluated the stiffness as very severe.

All the non-ambulatory participants with NM needed mobility aids and products in their daily lives. Six participants with NMr need orthoses (or other joint supports) and one of them uses crutches if necessary. Five ambulatory participants with NM use orthoses or mobility aid; one uses ankle orthoses, three need a walker or crutches, and one uses an electric scooter outdoors and for longer distances. Three ambulatory NM and two participants with NMr reported that they do not need a mobility aid or support (e.g., orthoses) at all. The older participants, however, more often reported difficulties in moving around in their homes, as well as the need of a mobility aid, especially outdoors. Participants aged 50 years or older reported using more products and technology for personal indoor and outdoor mobility compared with younger participants.

The non-ambulatory participants and two ambulatory participants with NM also reported having personal assistants and needing the help of other people in their daily living, but one reported that the help was not sufficient. Modifications of the home entrance had been made in six homes. Most of the participants, 80%, were satisfied with the health and social services. Of all participants, 65% received physiotherapy, two ambulatory participants with NM had applied and thought they would need physiotherapy, but it had not been granted, while five participants (2 NM and 3 NMr) felt they did not need physiotherapy at all.

Of the responders, 20% reported seasonal features, especially snow and ice, causing a lot of difficulties, or that they were unable to move outdoors, at all, during snowy or icy periods. The non-ambulatory persons had the most difficulties, and NMr patients reported experiencing more challenges than ambulatory NM patients. The percentages of responses according to their ICF block or category-based sum variables, as well as the means and standard deviations of the responses, are shown in Additional file 2(a).

### Self-reported psychological functioning

The differences in self-reported psychological functioning between the groups (NMr, ambulatory, and non-ambulatory NM) were not as big as in physical functioning (Fig. 2A), and the individual’s responses were more scattered across the groups. Yet, non-ambulatory participants reported more fatigue and emotional distress. All the participants were satisfied with their ability to do things at home for pleasure, such as reading or listening to music. The percentages of responses according to their ICF block or category-based sum variables, as well as the means and standard deviations of the responses, are shown in Additional file 2(b).

### Social functioning and satisfaction with social activities and roles

The responses to items addressing satisfaction with the ability to participate in social life and roles were quite evenly distributed from 1 (very satisfied) to 4 (a little bit satisfied) in all groups (Fig. 2a). One of the non-ambulatory participants’ responses, however, indicated not being satisfied with their social life at all. Half of the participants either studied or worked full-time, while one worked part-time, and all were at least somewhat satisfied with their capability to do work considered important by the responder. Work in this context includes both remunerative and non-remunerative. All non-ambulatory persons and two other participants had been granted disability pensions, but 83% of them were quite satisfied with their ability to do, for example, voluntary work or running a small business part-time. Only one, non-ambulatory participant, was not satisfied at all with her capability to do work. The percentages of responses according to their ICF block or category-based sum variables, as well as the means and standard deviations of the responses, are shown in Additional file 2(c).

### Impact of the COVID-19 pandemic on the participants’ self-reported functioning

The COVID-19 pandemic affected the physical functioning, general health, and daily living of ambulatory participants on average only a little. In contrast, the non-ambulatory participants reported that the pandemic affected them severely or even very severely. They also found that the pandemic restricted their access to social and health services more than the ambulatory participants did (Fig. 2b). Non-ambulatory participants felt that the pandemic had affected their emotional well-being more negatively, disturbed their sleep more and increased their fatigue compared with ambulatory persons. The younger and non-ambulatory participants reported more often that the COVID19 pandemic caused social and mental suffering than did older and ambulatory participants; they also avoided seeking health and social services due to fear of infection. The percentages of responses according, the mean values and standard deviations to their ICF blocks or categories are shown in Additional file 2(d).

None of the participants had had the infection by February 2022, when the study was completed.

### PROMIS T-score-based functioning of the participants compared with the reference population

**Fig. 3:**
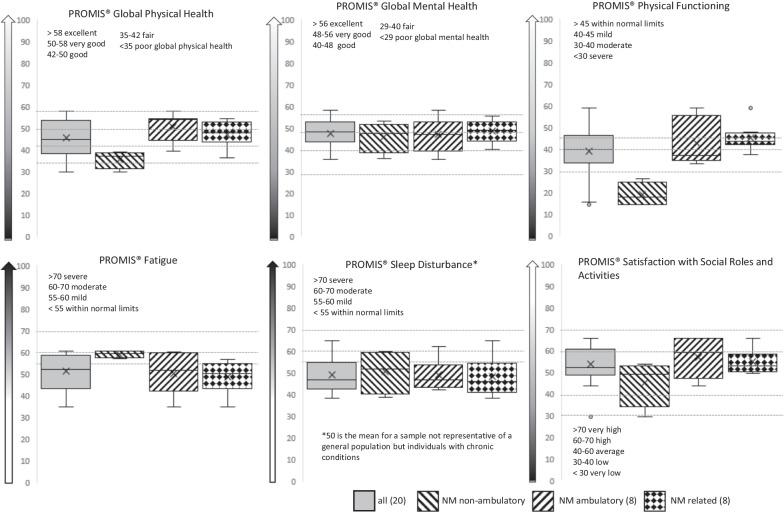
PROMIS® T-score based functioning of the non-ambulatory and ambulatory nemaline myopathy (NM) patients, and the participants with related disorders

The average functioning of the study participants was mostly good or within normal limits compared with the reference population, and the same trend was seen in the different areas of functioning (Fig. 3). In the instruments assessing global physical health and physical functioning, the non-ambulatory participants scored clearly lower than the ambulatory participants with NM or NMr. In addition, their score for fatigue was higher, while the T-scores for global mental health and sleep disturbances were very similar between the groups. Satisfaction with social roles and activities did not show clear differences between the patient categories, but the individual differences were more visible (Fig. 3). The percentages of the responses, mean T-scores for the groups and the ranges are shown in Additional file 3.

## Discussion

This survey showed that non-ambulatory participants of this study experienced more difficulties in their daily lives than ambulatory participants, and ambulatory participants with NM reported facing more challenges than participants with NMr, with a few exceptions. Non-ambulatory participants reported more conditions not directly caused by their muscle disorder as well as more co-morbidities likely secondary to their muscle weakness. According to the responses, asthma or other pulmonary diseases had been diagnosed by a medical doctor in (8) 40% of the participants. All of them used medication for it, and four of them had nocturnal respiratory support. It is, however, to be noted that breathing problems related to muscle weakness may easily be mistaken for asthma, because the slowness in expiration caused by muscle weakness is like that caused by bronchial constriction. Joint-related problems were most common in ambulatory participants with NM while these problems were rarer among wheelchair users and participants with NMr. Ambulatory participants with NM reported having more joint problems, such as hypermobility, stiffness and joint deformities compared with participants with NMr and non-ambulatory participants, which, in turn in combination with walking (and mobility in general) may have led to their having more arthritis and pain of the joints. In addition, compared to participants with NMr, ambulatory participants with NM had fewer joint supports or orthoses in use.

The non-ambulatory persons reported that changing and maintaining body position, arm and hand use, selfcare, mobility without aids, and doing housework was difficult or impossible, and that the climate and weather affected their ability to function. The ambulatory participants reported no or little difficulties performing daily tasks requiring some physical functioning, but heavier tasks were more challenging for ambulatory participants with NM than participants with NMr. This is in line with the clinical phenotypes—patients with NM have more generalized and proximal muscle weakness while patients with NMr have fewer, mainly distal muscles affected. The individual differences between the ambulatory participants with NM and NMr were great—some persons described their physical functioning as being even very good.

Gender, age, quality of sleep as well as coping skills have been proposed to affect the quality of life and functioning of people with muscle disorders [2]. In this series of patients, these factors did not, however, show any clear association with the self-reported emotional or psychological, nor physical functioning.

The family status, residency (large or small town), degree of education or mutated gene did not seem to be associated with the level of functioning. The type of pathogenic variant in *NEB*, however, affects the severity of the muscle weakness and subsequently affects the physical functioning of the person [11].

Although the non-ambulatory persons were not as satisfied as the ambulatory participants with their social life and emotional or mental functioning, sleep, and fatigue, the differences in these variables were smaller between the groups than in other areas of functioning, and the individual differences were bigger within all the groups. The non-ambulatory participants also reported more fatigue that the ambulatory ones. COVID-19, however, affected the well-being of non-ambulatory participants more than that of others, also affecting their access to social and health services more.

PROMIS T-scores show that, on average, the functioning of the participants in this study is close to that of the reference population. Physical functioning, sleep disturbances, and fatigue, as well as global health, are likely to be experienced similarly in Western countries such as Finland, or the US. Satisfaction with social life and activities, however, might be more affected by cultural differences. It was also notable that in the ICF classification, leisure time was linked to social life, or spending time with other people. Reviewing the mean T-scores of the entire patient group, the scores were on average slightly lower than the T-scores of the reference population, but better in Sleep Disturbances and Satisfaction with Social Life and Activities. This could be due to cultural differences, but another possibility is that people with disabilities might have adapted to their situation, thus not experiencing their disability as limiting their functioning to the extent that might have been expected [42]. Like people without disability, people with disabilities have individual backgrounds, personalities, and environments, all affecting their psychological resilience and coping skills, and subsequently their functioning and well-being also [43]. The results of this study showed that participants with NM form a more functionally heterogenous group than participants with NMr, who in general had milder muscle weakness. Some NM participants with mild forms, however, had even less impaired functioning than participants with NMr. Ambulatory participants of this study had better functioning than wheelchair users when physical functioning was measured, while there were no differences in the self-reported psychological functioning. Social functioning was better among ambulatory participants. Age was not strongly associated with functioning. This might be explained by the fact that the clinical phenotype varied in all age groups. It should, however, be noted that the participants using wheelchairs were between 22 and 60 years of age, while the eldest participants in the study were 75 years of age. It may be concluded that in this study, the clinical phenotype probably affected the physical phenotype more than did the age.

Compared with ambulatory participants, the social contacts as well as the frequencies of non-ambulatory participants in attending physiotherapy and other health and social services were reduced more due to the COVID-19 pandemic. This was either due to difficulties to access the services or due to fear of infection. This study indicates that people with disabilities dependent on the help of others and in a need of rehabilitation (e.g., physiotherapy) are particularly vulnerable during global or national exceptional periods. An action plan to ensure continuous rehabilitation and assistive services should be developed for any similar periods in the future. In addition, opportunities for social contacts should be ensured.

There has been little research on self-reported functioning of adult persons with ultra-rare muscle disorders. As the present one, the studies done hitherto are often either quite comprehensive and concern a specific disorder(s) with a small number of participants, or more specific, investigating a few factors affecting the functioning or quality of life in a larger sample of participants with different disorders. The methods used vary between studies and are tailored to meet the subject and the target group studied [26, 44]. In general, the quality of life of people with muscle disorders in all areas of functioning has been reported to be lower than that of the healthy population, but between disorders, the differences have been statistically small [1]. It was shown already two decades ago that the severity of a physical disability does not automatically alone reduce the quality of life [42]. Many factors, however, seem to strongly affect the lived experience of functioning and the quality of life. A few cross-sectional studies have shown that while muscle weakness does affect the physical functioning and subsequently also the performing of tasks requiring functional muscles, the degree of fatigue and pain affects the quality of life. Also, impairments in mental functions, pain and restrictions in participation in life situations (social functioning) have a stronger association with quality of life. Pain, however, is not a common symptom in NM or NMr, which was notable in this survey, too. The severity of the muscle disorder does seem to be associated with lower social functioning, likely due to the more restricted abilities to participate in social events or situations. Surprisingly however, this does not seem to affect psychological functioning [1–4, 44].

### Limitations

The present study was a pilot study, and therefore one of the aims was to test the survey and the methodology for future studies. The survey was not optimally dimensioned in some subject areas. Most of the items were from PROMIS item bank, and a few items were either from other studies or self-designed. We approached the functioning from two different angles: using both ICF categories and T-scores of the selected PROMIS instruments. The T-scores allow comparison with the PROMIS reference population, which consisted of U.S. residents, as no reference T-scores are available for the Finnish population. As the items of PROMIS instruments have been designed to review the same subject, using T-scores is more straightforward and can be calculated directly from the responses using a tool provided by the PROMIS measuring system. All the items on the 5-point Likert scale were linked to ICF categories by two researchers, and researchers of some other study might have linked the same items to different categories. Linking some PROMIS items to ICF categories is challenging and one item could often fit into two or three ICF categories. In addition, the items of one PROMIS instrument might all be linked to different ICF categories. However, this enables the utilization of the globally understood ICF system and dissection of the different areas of functioning. As we did not have a control group, we compared clustered sub-groups of the cohort. As for many studies on ultra-rare disorders, the small study sample did not allow for meaningful statistical analyses. Therefore, further international studies and a larger number of responders are needed for credible statistical analyses. If should be borne in mind that the results of this study concern a small patient cohort resident in one country and cannot be supposed to be global, but the current results point to possible differences which might be found when the study is repeated in larger patient sample.

## Conclusions

The pilot study we conducted in 20 Finnish persons with NM and NMr showed that they form a heterogeneous group of people with large interindividual differences in functioning in their daily lives. Some needed personal assistance in most of their daily tasks, daily routines, and self-care, while the muscle weakness of other participants was so mild or affected only specific muscles, so that they did not experience the disorder as lowering their ability to function in their everyday lives. Responders who could walk with or without a mobility aid had better physical functioning than wheelchair users, but there were no differences in the self-reported psychological functioning. Exceptional situations, such as pandemics, seem to affect the lives of people with disabilities more than their non-disabled peers. Especially vulnerable are those using wheelchairs and those who are dependent on assistance in their daily lives.

### Future directions

Our future aim is to extend the study internationally to reach a larger number of NM patients and to include other congenital muscular disorders as well, utilizing a modified survey that has been improved based on our experience from this pilot study. The existence of individual differences in the one’s experience of functioning in daily life should be borne in mind and the possible experiences of an individual should not be presupposed when medical care, rehabilitation, or other services for people with disabilities are planned.

### Supplementary Information

**Additional file 1**: ICF categories for the areas of functioning assessed; the source of the item used (and number of items), and Cronbach’s α-value for the sum variables.

**Additional file 2 A–D**: Percentages of responses according to their International Classification of Functioning, Disability and Health (ICF) category-based sum variables (the number of summed items).

**Additional file 3**: The mean values and ranges of T-values for the PROMIS-instrument based functioning. The levels of functioning defined by the Promis Health Organisation and the percentages show the proportion of the patient's level in each group.

## Data Availability

The data generated (responses of the participants) and analyzed are not publicly available due to individual privacy but are available as anonymous from the corresponding author. Based on the Health Measures Terms of use, a clean copy of the questionnaire is not allowed to be included in the manuscript because it includes PROMIS questions. All the PROMIS instruments and items are available in English at www.healthmeasures.net.

## Abbreviations

NM: Nemaline myopathy
NMr: Nemaline myopathy-related disorder
*NEB*: Nebulin gene
*TPM2*: Gene encoding alpha-tropomyosin
PROMIS^®^: Patient Reported Outcomes Measurement Information System
ICF: Classification of Functioning, Disability and Health
LYHTY: Lyhytkasvuisten toimintakyky ja haasteet esteettömyydessä ja yhdenvertaisuudessa -tutkimus (The functional capacity of people of short stature and the challenges they face with accessibility and equality)
