# Supplementary material for: Self-reported functioning among patients with ultra-rare nemaline myopathy or a related disorder in Finland: a pilot study
Source: Orphanet J Rare Dis. 2023 Nov 30;18:374. doi: 10.1186/s13023-023-02973-2 (PMC10691147; doi:10.1186/s13023-023-02973-2)
Supplement: Supplementary file 1 — Additional file 1: ICF categories for the areas of functioning assessed; the source of the item used (and number of items), and Cronbach’s α-value for the sum variables. [file 13023_2023_2973_MOESM1_ESM.pdf]

Additional file 1: ICF categories for the areas of functioning assessed; the source of the item used (and number of items), and Cronbach's α-value for the sum variables.

b: BODY FUNCTIONS (FNCS)

b1: MENTAL FUNTIONS

b130 energy and drive fncs (**fatigue**)
PROMIS® - Fatigue (4)
PROMIS® - Global health (1)
α-value 0.908

b134 sleep fncs:
PROMIS® - Sleep Disturbance (4)
α-value 0.906

b152 emotional fncs:
PROMIS® - Global Health (1)
PROMIS® - Anxiety (1)
PROMIS® - Anger (1)
PROMIS® - Depression (3)
α-value 0.894

b2: SENSORY FNCS AND PAIN

b280 sensation of **pain**
PROMIS® - Global Health (1)
PROMIS® - Pain Interference (3)
α-value 0.937

b2801(a) joint pain
LYHTY (1)

b2801(b) muscle pain
LYHTY (3)
α-value 0.829

b7: NEUROMUSCULOSKELETAL AND MOVEMENT-RELATED FNCS

b735 muscle tone fncs
LYHTY (2)
α-value 0.732

d: PARTICIPATION AND ACTIVITIES

d2: GENERAL TASKS AND DEMANDS

d230 carrying out daily routine
PROMIS® - Satisfaction with Participation in Social Roles (PSR) (1)

d4: MOBILITY

d410 changing and maintaining body position
PROMIS® - Physical Functioning (5)
RG (1)
α-value 0.947

d445 hand and arm use
PROMIS® - Physical Functioning (8)
α-value 0.958

***d450 Walking and D465 moving around using equipment**
RG (3)
α-value 0.95

d4600 moving around within a home
PROMIS® - Physical Functioning (1)

d5: SELFCARE

d510 washing oneself, D530 toileting, d540 dressing
PROMIS® - Physical Functioning (7)
α-value 0.959

d6: DOMESTIC LIFE

d620 acquisition of goods and services
PROMIS® - Physical Functioning (2)
α-value 0.79

d640 doing housework
PROMIS® - Physical Functioning (1)

d698 other specified domestic life
-**leisure time at home**
PROMIS® - Satisfaction with Participation in Discretionary Social Activities (DSA) (1)

d7: INTERPERSONAL INTERACTIONS AND RELATIONSHIPS

d7500 relationships with **friends** and d760 **family**
PROMIS® - Satisfaction with PSR (1)
PROMIS® item - Satisfaction with Participation in DSA
(1) α-value 0.87

d8: MAJOR LIFE AREAS

d850 remunerative and **d855** non-remunerative **employment**
PROMIS® - Satisfaction with PSR (1)

d9: COMMUNITY SOCIAL AND CIVIC LIFE

d920 Recreation and leisure
PROMIS® - Satisfaction with Participation in DSA (1)

e: ENVIRONMENTAL FACTORS

e1: PRODUCTS AND TECHNOLOGIES

e1151 assistive products and technology for personal use in daily living
LYHTY (1)

***e1201 assistive products** and technology **for personal indoor and outdoor mobility** and transportation
RG (3)
α-value 0.95

e2: NATURAL ENVIRONMENT

e225: climate
LYHTY (2)
α-value 0.723

e298 other specified natural environment
Covid-19 pandemic
e298 (impact on):
- fatigue
- sleep
- emotions
- pain
- general health
- physical functioning
- daily living
- social life
- applying to health and social services due to the fear of infection
- access to health and social services

*****d450 Walking and d465 moving around using equipment and e1201 assistive products and technology for personal indoor and outdoor mobility were addressed in same questions.

ICF: International Classification of Functioning, PROMIS®: Patient-Reported Outcomes Measurement Information System
